# Refining the stress gradient hypothesis for mixed species groups of African mammals

**DOI:** 10.1038/s41598-022-22593-3

**Published:** 2022-10-21

**Authors:** Christian Kiffner, Diana M. Boyle, Kristen Denninger-Snyder, Bernard M. Kissui, Matthias Waltert, Stefan Krause

**Affiliations:** 1 Junior Research Group Human-Wildlife Conflict and Coexistence, Leibniz Centre for Agricultural Landscape Research (ZALF), Müncheberg, Germany; 2 The School for Field Studies, PO Box 304, Karatu, Tanzania; 3 Department of Conservation Biology, Georg-August Universität Göttingen, Göttingen, Germany; 4 Department of Fish, Wildlife, and Conservation Biology, Colorado State University, Fort Collins, USA; 5 Grumeti Fund, PO Box 65, Mugumu, Tanzania; 6 Lübeck University of Applied Sciences, Lübeck, Germany

**Keywords:** Ecology, Behavioural ecology, Community ecology, Ecological networks, Evolutionary ecology

## Abstract

Species interactions such as facilitation and predation influence food webs, yet it is unclear how they are mediated by environmental gradients. Here we test the stress gradient hypothesis which predicts that positive species interactions increase with stress. Drawing upon spatially-explicit data of large mammals in an African savanna, we tested how predation risk and primary productivity mediate the occurrence of mixed species groups. Controlling for habitat structure, predation risk by lions and primary productivity affected the frequency of mixed species groups in species-specific ways, likely reflecting distinct stress perceptions. To test whether mixed species groups indicate positive interactions, we conducted network analyses for specific scenarios. Under predation risk, dyadic associations with giraffes were more pronounced and metrics of animal networks changed markedly. However, dyadic association and network metrics were weakly mediated by primary productivity. The composition of mixed species groups was associated with similarities in prey susceptibility but not with similarities in feeding habits of herbivores. Especially predation risk favoured the frequency of mixed species groups and pronounced dyadic associations which dilute predation risk and increase predator detection. While our results provide support for the stress gradient hypothesis, they also highlight that the relative importance of stressors is context-dependent.

## Introduction

A fascinating phenomenon in organismic biology is the association of different species^1–3^, with mixed species groups (MSGs) of large mammal species in African savannas being a prominent example. Individuals from different species may associate for several reasons, and variation in predation risk and resource availability have been hypothesized to mediate the frequency and structure of MSGs^4–8^. More recently, the formation of MSGs has been linked to the stress gradient hypothesis (SGH)^9^. The SGH postulates an increase in positive species interactions with increasing environmental stress^10,11^. Originating in plant community ecology, this hypothesis has recently been tested among animal communities^12,13^. The underlying mechanisms for the SGH are that interacting species can buffer one another from potentially limiting physical stressors^11^. In the context of large herbivore populations in African savannas, predation pressure by large carnivores and resource scarcity are key stressors^14^, and both variables have been put forward as hypotheses to explain occurrence of MSGs^4–7^.

Predation risk is a strong evolutionary factor that affects multiple aspects of behaviour in species^15,16^, and prey species typically perceive variation in predation risk and adjust their behaviour in a “landscape of fear”^17–19^. A key response of prey species to variation in predation risk is to alter aggregation patterns by adjusting their group size or composition^20^. Available evidence suggests that mammals in mixed groups benefit from complementary modes of predator detection and effective information transmission across species boundaries and from diluted per capita predation risk^21^. Thus, if aggregating with other species has fitness relevant benefits, the SGH predicts that prey species associate with other species in areas where they are subject to greater predation risk. While this prediction has been supported in quantitative terms, exemplified by MSGs of grazing herbivores more likely to occur in areas with high lion (*Panthera leo*) predation risk^9^, we additionally predict that species associations change in composition. Such structural changes could arise in areas with high predation risk, if species associate with heterospecifics with complementary modes of predator detection to increase predator detection or with a similar susceptibility to predation to dilute individual risk.

Beyond reducing predation risk, associating with other species may provide benefits in terms of localizing and acquiring food resources, for example via heterospecific information transfer^21,22^. If animal associations followed predictions of the SGH, we would expect that MSGs form most frequently under conditions of resource scarcity. However, quantitative tests of the SGH within animal communities did not always support this prediction^13^ and in East African herbivore communities, species associations were often positively associated with primary productivity^7,9^. While these patterns may speciously contradict predictions of the SGH, the directionality of stress associated with primary productivity may likely differ across species and could be mediated by body size and digestive strategy^23^, but Ref.^24^. For example, small-bodied herbivore species such as Thomson’s gazelle (*Eudorcas thomsonii*) should forage in patches of high quality forage, and avoid patches of high primary productivity as stressful. This is because primary productivity is often inversely associated with forage quality due to low concentration of nutrients and low leaf:stem ratios in highly productive vegetation tissue^25^. Additionally, small-bodied species may have difficulties in assessing tall vegetation and possibly also fear predation risk in tall grass patches^26^. In contrast, larger-bodied species, and species with inefficient digestive systems, may perceive areas of low primary productivity as stressful as they may not meet their quantitative nutritional demands in low-productivity patches^27^. Thus, while feeding habits of many herbivore species in African savannas overlap^28,29^, and energy intake can be mediated by the interplay between forage quality, forage quantity, body size and digestive strategy, primary productivity could influence species co-occurrences in non-linear ways.

The distribution of African herbivore species is tied to the structural properties of the vegetation. Clearly, these habitat-associations could mediate the occurrence of MSGs^23^. However, because habitat-associations are species-specific (e.g. some species prefer grasslands, others prefer wooded areas), it is difficult to predict how habitat structure affects quantity and composition of MSGs.

Human activity can also cause stress to wildlife species. For example, recent evidence from the Serengeti ecosystem suggests that human activities affect the distribution of grazing herbivores via direct (e.g. illegal hunting) or indirect (e.g. changes in habitat structure and competition with livestock) pathways^30,31^. According to the SGH, we thus expected that herbivores were more likely to form MSGs in areas with greater human presence.

**Figure 1:**
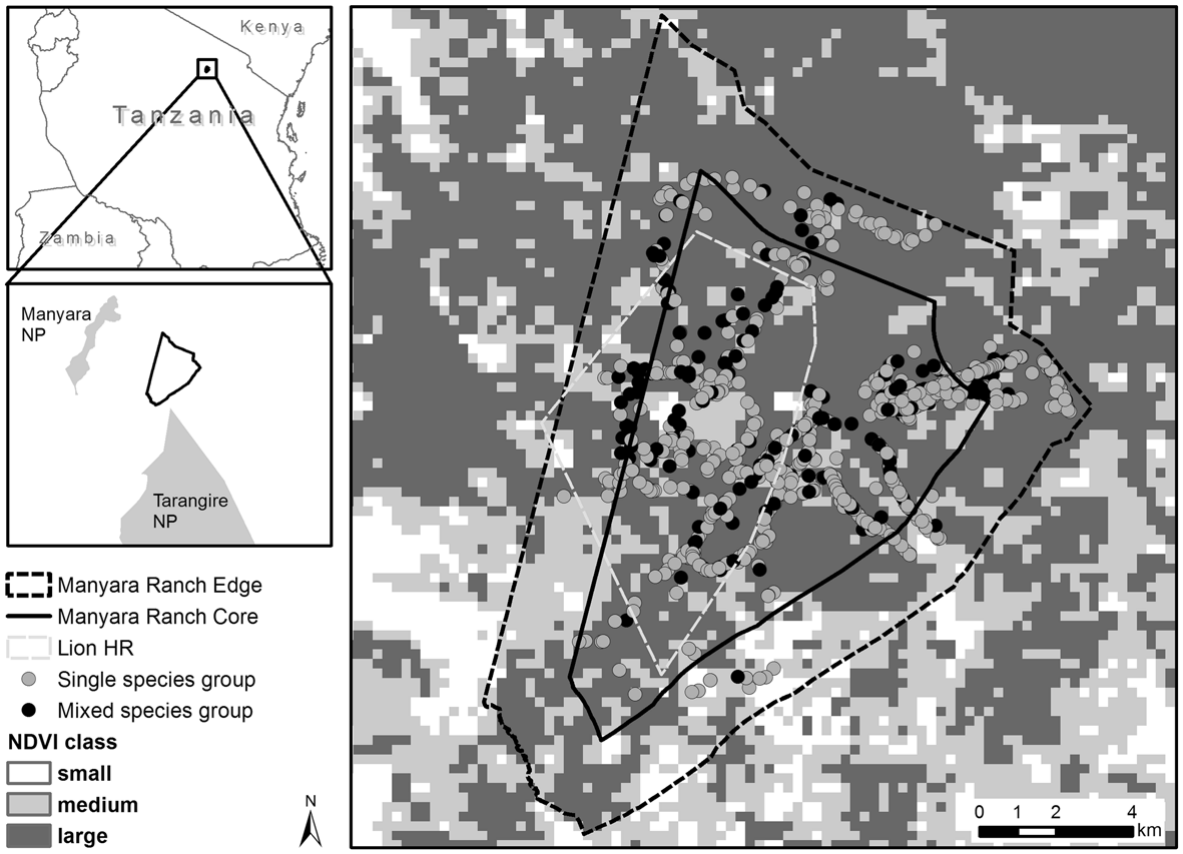
Location of single- and mixed-species groups of large mammal species, in relation to lion home range (85% minimum convex polygon) and vegetation productivity (mean NDVI from study period; darker shading corresponds with greater NDVI values) within Manyara Ranch, northern Tanzania.

Building upon work in the Serengeti ecosystem^9^, and drawing upon a species-rich mammal community dominated by large herbivores (Table 1) in a conservation area of northern Tanzania (Fig. 1), we here test the SGH from different angles and using different lenses. First, we test if the frequency of MSGs aligns with a suite of environmental stressors (Table 2): predation risk by lions (indicated by locations inside the lion home range, LHR; hypothesis H1), primary productivity (indicated by the Normalized Difference Vegetation Index, NDVI; H2), and human activity (indicated by spatial variation of land use intensity; H3). Since habitat structure may affect the distribution of species, we also controlled for this variable (indicated by a qualitative description of the vegetation physiognomy; H4). For these analyses, we first chose the community perspective to identify the overall effect of the hypothesised stressors on the frequency of MSGs (H1–H4). To account for species-specific stress effects^32^ we then shift the perspective to single species (focusing on the more abundant species; Table 1) and assess how predictions from H1 to H4 vary by species.

**Table 1:** Common and scientific names, as well as number of occurrences (N = 1254) of large mammal species observed in Manyara Ranch, Tanzania, listed in descending order of body mass.

Common name	Scientific name	Number of sightings	Jacobs’ index score	Proportion monocots in diet
Elephant	*Loxodonta africana*	14	− 0.87	0.23
**Giraffe**	***Giraffa camelopardalis tippelskirchi***	**128**	**0.24**	**0.05**
Buffalo	*Syncerus caffer*	1	0.32	0.78
Eland	*Taurotragus oryx*	34	0.18	0.50
**Zebra**	***Equus quagga***	**498**	**0.16**	**0.92**
**Wildebeest**	***Connochaetes taurinus***	**118**	**0.27**	**0.81**
Waterbuck	*Kobus ellipsiprymnus*	20	0.18	0.84
Warthog	*Phacochoerus africanus*	15	0.11	0.91
Lesser kudu	*Tragelaphus imberbis*	16	− 0.2	0.34
**Grant’s gazelle**	***Nanger granti***	**96**	− **0.56**	**0.65**
**Impala**	***Aepyceros melampus***	**137**	− **0.73**	**0.40**
Gerenuk	*Litocranius walleri*	2	− 0.73	0.00
**Thomson’s gazelle**	***Eudorcas thomsonii***	**85**	− **0.62**	**0.75**
Olive baboon	*Papio anubis*	2	− 0.89	0.20
Steenbok	*Raphicerus campestris*	6	− 0.86	0.34
Black-backed jackal	*Canis mesomelas*	9	− 1.00	0.06
Kirk’s dik–dik	*Madoqua kirkii*	57	− 0.88	0.17
Vervet monkey	*Chlorocebus pygerythrus*	16	− 1.00	0.07

For each species, we also include the Jacobs’ index which quantifies the prey preference (positive values) or avoidance (negative values) by lions^68^, and the percentage of monocot consumption^28,69–73^. For the six most abundant species (highlighted in bold), we conducted species-specific analyses.

**Table 2:** Testing the stress gradient hypothesis for mixed species groups (MSG) of African savanna herbivores.

	Hypothesis	Prediction	Test
**Quantitative aspects of MSGs**			
H1	Predation risk mediates MSG occurrence	Greater likelihood for MSG occurrence inside the lion home range (LHR) vs. outside of the LHR	Logistic regression
H2	Primary productivity mediates MSG occurrence	Since primary productivity is essential for herbivores, we expected a strong signal of this variable. However, because stress perceptions associated with primary productivity (indicated by NDVI) varies by species (i.e. small bodied species generally perceive tall grass [which could be indicated by high NDVI conditions] as stressful due to low nutrient concentration and high predation risk, whereas large bodied species may perceive low NDVI as stressful due to their food quantity requirements), a prediction on the general direction of its effect is not possible	Logistic regression
H3	Human-induced stress mediates MSG occurrence	Lower likelihood for MSG occurrence in the core area of the ranch vs. the edge area of the ranch	Logistic regression
H4	Habitat structure mediates MSG occurrence	No specific prediction; will depend on habitat preferences of each species	Logistic regression
**Structural and compositional aspects of MSGs**			
H5	If the frequency of MSGs is mediated by environmental variables (H1–H4), we would also expect the structure of animal networks to differ across environmental conditions	Under environmental conditions favoring the frequency of MSGs (H1–H4), we would expect (1) that species generally increase the strength of associations with heterospecifics (increase in *Node strength*), (2) that the variability of species associations varies (changes in *Y-measure*), and that (3) the transitivity of associations increases, i.e. the tendency of species to form highly connected subsets increases (increase in *Clustering coefficient*)	Welch’s t-test on aggregated values of subsampled networks
H6	We expected dyadic associations to emerge under stressful conditions	Under stressful conditions we expected to observe additional dyadic associations to emerge, which cannot be explained by chance alone	Randomization test
H7	Prey preferences of lions and food preferences of herbivores mediate the composition of MSGs	Species with similar susceptibility to lion predation are more likely to be found together in MSGs in order to reduce predation risk; species with similar food preferences are more likely to occur together in MSGs because these species may provide information on resource availability	Randomization test

For each specific hypothesis, we outlined predictions and indicated the type of test (see “Methods” section for more details).

To test the SGH in terms of structural properties of the MSGs, we analysed animal networks for those environmental conditions that influenced the frequency of MSGs (Table 2). If environmental stressors favoured MSG formation (conditional on results regarding H1–H4) we would expect that properties of animal networks changed accordingly (H5). Moreover, we would expect that additional dyadic associations emerged under stressful conditions (H6). If predation risk was the key driver for MSG formation, we would expect that similarity in susceptibility to lion predation explained the composition of MSGs. If, however, information about food availability was the main driver for MSG composition, we would expect that similar feeding habits of herbivores explained the composition of MSGs (H7).

## Results

### Occurrence of mixed species groups

**Figure 2:**
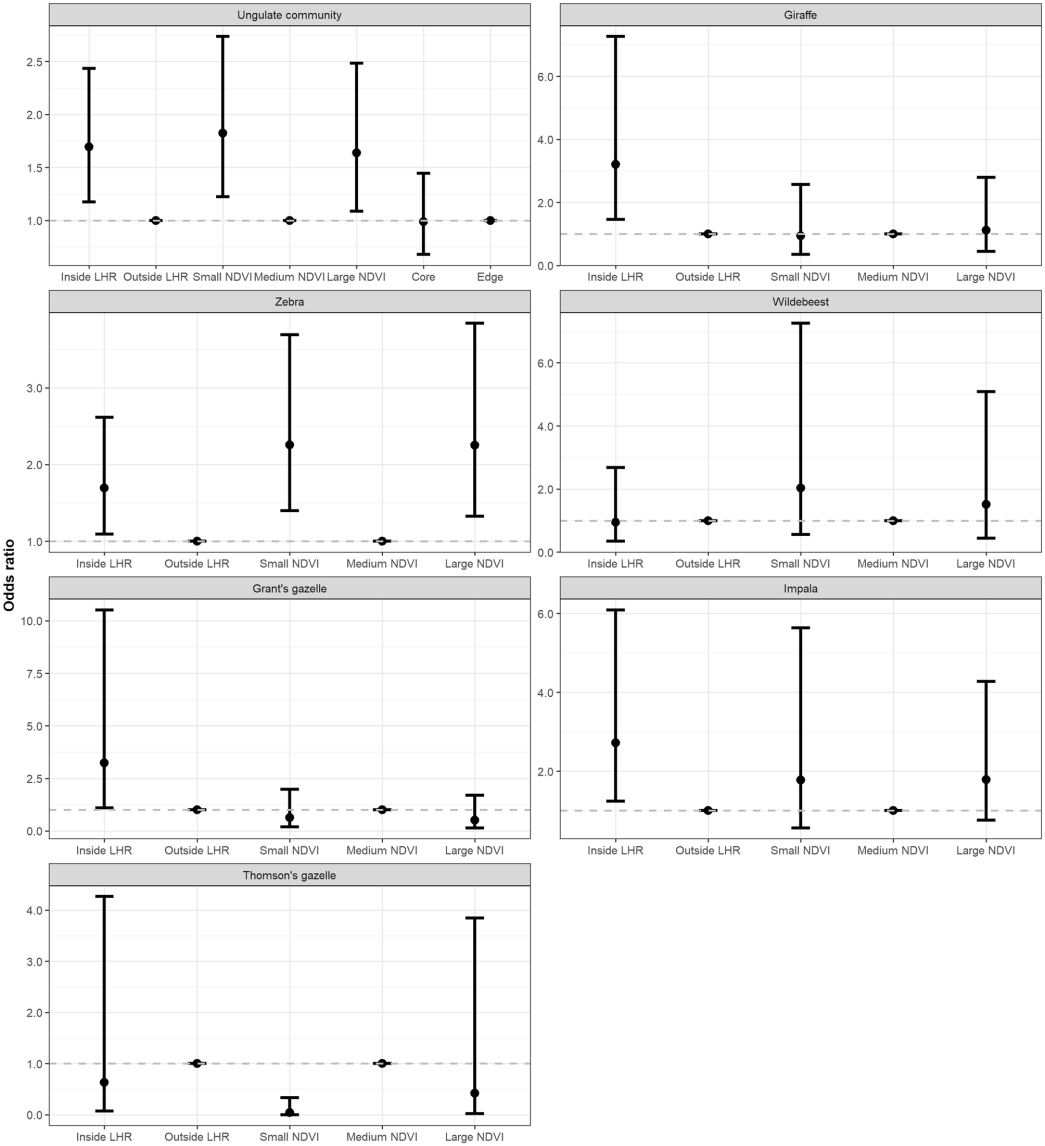
Odds ratios depicting the relative effects of primary productivity (indicated by small and large NDVI values relative to medium NDVI values) and predation risk (indicated by location of the observation inside vs. outside the lion home range [LHR]) on the likelihood of mixed species group occurrence in the entire ungulate community and separately for six species within Manyara Ranch, northern Tanzania.

To test if environmental stressors affected the frequency of MSG occurrence (Table 2: H1–H4), we fitted logistic regression models testing the effect of all four explanatory variables (collinearity across explanatory variables was negligible; Table S1). For the entire set of animal groups (n = 951), MSGs were 1.49 (95% CI 1.04–2.17) times more likely to occur in open bushland compared to closed bushland, 1.82 (1.22–2.74) and 1.64 (1.09–2.48) times more likely to occur in areas with small and large NDVI values, respectively (both compared to areas with medium NDVI values). MSGs were 1.69 (1.18–2.43) times more likely to occur inside the LHR compared to areas outside (Table S2, Fig. 2). Because the variable that described the human activity (location in the edge area or core area of the ranch) did not markedly affect the frequency of MSGs at the community level [odds ratio: − 0.012 (95% CI − 0.387–0.369), p = 0.949], we omitted this variable from species-specific models.

From the perspective of single species, the strength and direction of associations between MSG-occurrence and environmental variables varied (Fig. 2, Table S2). Giraffe groups were 3.21 (1.46–7.26) times more likely to occur in MSGs when encountered inside vs. outside the LHR. Compared to areas with medium NDVI, zebra groups were 2.26 (1.40–3.69) and 2.25 (1.33–3.84) times more likely to occur in MSGs in areas with small and large NDVI values, respectively. Inside the LHR, zebra groups were 1.69 (1.10–2.62) times more likely to occur in MSGs. In wildebeest, none of the tested variables produced a significant effect. The occurrence of MSGs in Grant’s gazelles and impala was greater in open bushland than in bushland [Grant’s gazelle: 3.46 (1.18–10.86); impala: 2.27 (1.00–5.20)] and greater inside vs. outside the LHR [Grant’s gazelle: 3.24 (1.10–10.51); impala: 2.72 (1.23–6.09)]. Compared to areas with medium NDVI values, Thomson’s gazelles were 0.04 (0–0.33) times less likely to occur in MSGs in areas with small NDVI values.

In sum, LHR, NDVI, and habitat structure mediated the frequency of MSG, thus largely supporting H1, H2, and H4. However, we found no evidence for the human activity variable to affect the frequency of MSG (H3).

### Animal networks

**Figure 3:**
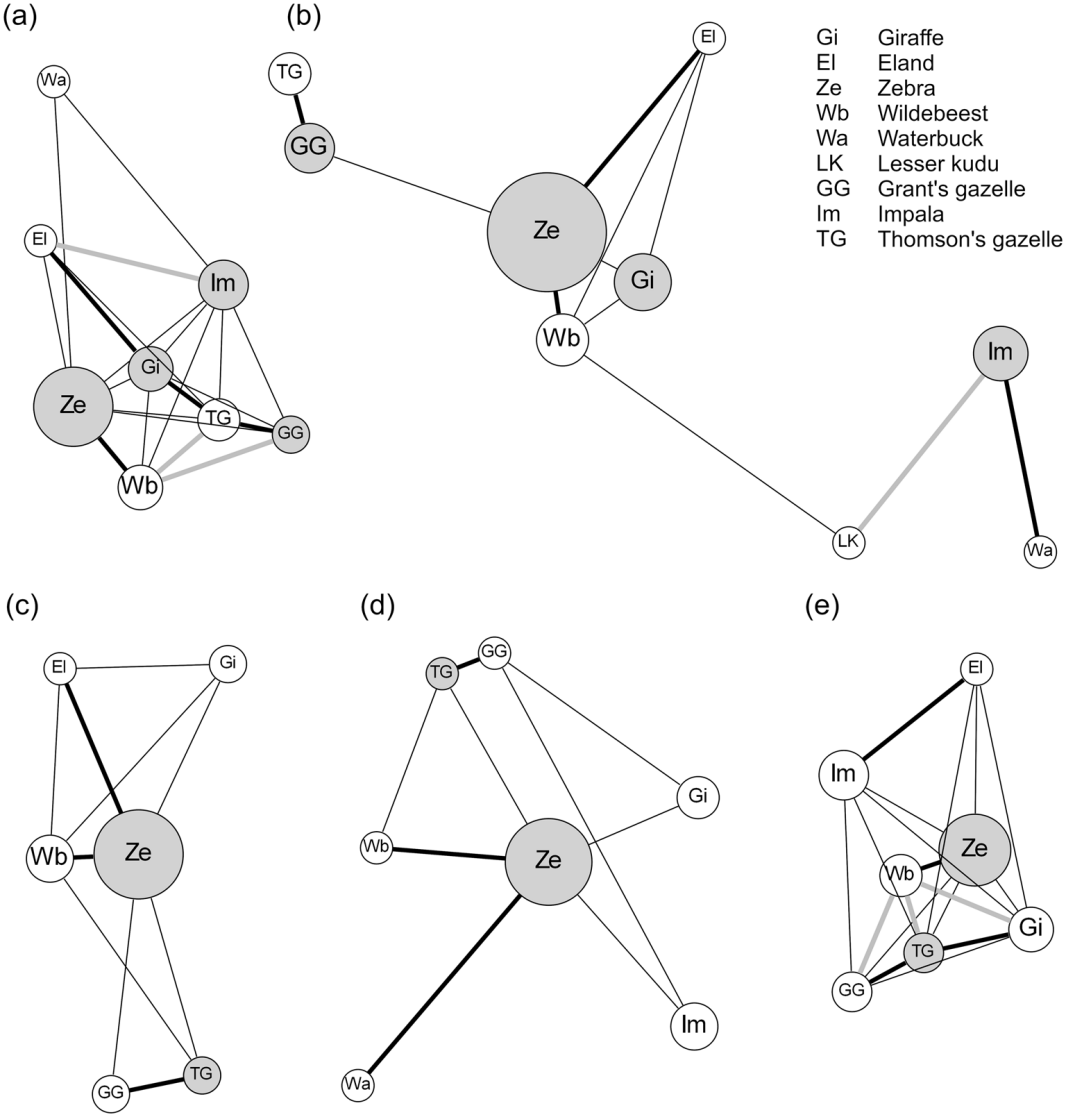
Social networks of focal species (grey circles) and non-focal species (white circles) based on animal groups observed (**a**) inside and (**b**) outside lion home range, and under (**c**) small, (**d**) medium and (**e**) large NDVI conditions. The thick black lines indicate associations that are significant, even if preferences for the same habitat types are taken into account, while for the thick grey lines the significance can be explained by such preferences. The node areas are proportional to the observed numbers of occurrences of the species. The layout follows a spring model, where the lengths of the edges correspond to the association strengths (Dice’s index). A short edge indicates a high value. For better readability, an edge is only shown, if its weight was ≥ 0.05 and if the two species co-occurred at least twice. Similarly, only the focal species and those species are shown that were connected by such an edge. However, the spring layout takes all edges into account. The networks were drawn using Graphviz version 2.38 (http://graphviz.org/).

To investigate the SGH in terms of structural properties of the MSGs, we described animal networks for the different environmental conditions. Corresponding to the results regarding the two key environmental stressors (predation risk and primary productivity), Fig. 3 illustrates the roles of zebra, impala, giraffe, and Grant’s gazelle in the networks inside and outside the LHR (Fig. 3a,b), and of zebra and Thomson’s gazelle in the networks for areas with small, medium and large NDVI (Fig. 3c–e).

**Figure 4:**
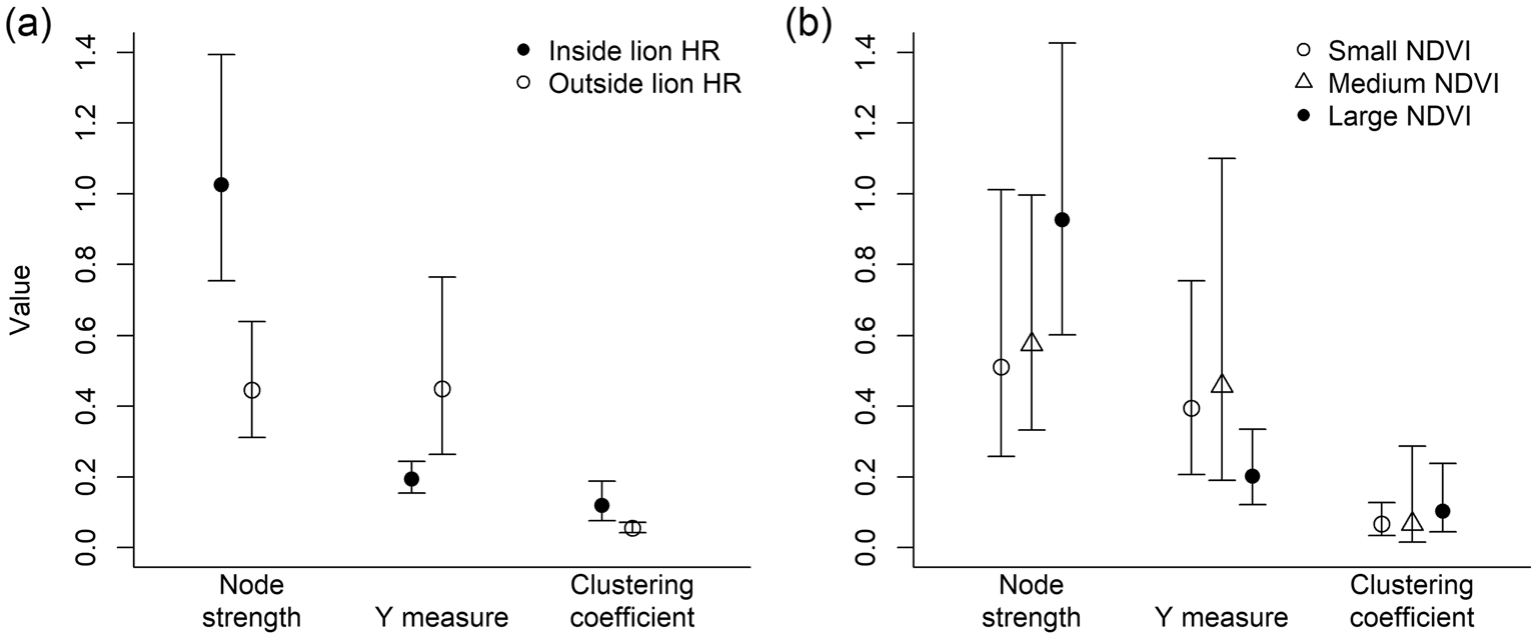
Means and 95% confidence intervals of measures for 3 independent subnetworks of (**a**) the networks inside and outside lion home range, and (**b**) the networks under small, medium, and large NDVI conditions.

The networks inside and outside the LHR differed significantly regarding each of the 3 investigated network measures (Fig. 4, Table S3). Inside the LHR, the node strength (*t* = 7.58, *p* = 0.002) and the weighted clustering coefficient (*t* = 6.42, *p* = 0.006) were greater than outside, while the *Y*-measure was smaller (*t* =  − 6.25, *p* = 0.011). The networks for small and medium NDVI did not differ for any measure (all *p*-values > 0.59). However, both of them had a smaller node strength than the network for large NDVI (small versus large NDVI: *t* =  − 3.17, *p* = 0.043; medium versus large NDVI: *t* =  − 2.93, *p* = 0.046) and a smaller *Y*-measure (small versus large NDVI: *t* = 3.52, *p* = 0.027; medium versus large NDVI: *t* = 3.48, *p* = 0.036), while there was no significant difference regarding the weighted clustering coefficient (small versus large NDVI: *t* =  − 1.78, *p* = 0.154; medium versus large NDVI: *t* =  − 1.10, *p* = 0.349).

In line with H5, the structure of animal networks was markedly different when comparing scenarios with and without predation risk by lions. However, variation in primary productivity did not markedly affect network metrics.

### Association patterns

To assess if specific dyadic associations emerge only under stressful conditions, we used a comparative approach while accounting for differences in habitat structure. Although for several species the likelihood to occur in a MSG differed between the 5 networks, the numbers of significant species-specific associations that could not be explained by preferences for habitat types did not differ substantially (Fig. 3, Table 3). In all 5 networks we found significant associations between zebra and wildebeest and between Grant’s and Thomson’s gazelles. Inside the LHR, giraffes were associated with Thomson's gazelles and elands; under large NDVI conditions they were associated with Thomson's gazelles. Several associations inside the LHR and under large NDVI conditions between wildebeest and other species were only significant, if the randomisations did not take the habitat type into account (Table 3).

**Table 3:** Dice indices for species dyads and results of randomisation tests (10^7^ steps; described in the “Methods” section) testing the strengths of dyadic associations observed in the five networks.

Network	Pair of species		Dice’s index	p-values, if preferences for habitat types are	
				Not considered	Considered
Inside lion home range	Zebra	Wildebeest	0.28	< 0.001	0.001
	Thomson’s gazelle	Grant’s gazelle	0.34	< 0.001	0.001
	Thomson’s gazelle	Giraffe	0.22	0.008	0.030
	Giraffe	Eland	0.13	0.016	0.035
	*Wildebeest*	*Thomson’s gazelle*	*0.24*	*0.003*	*0.079*
	*Wildebeest*	*Grant’s gazelle*	*0.17*	*0.046*	*0.351*
	*Impala*	*Eland*	*0.11*	*0.029*	*0.080*
Outside lion home range	Zebra	Wildebeest	0.26	< 0.001	< 0.001
	Zebra	Eland	0.07	0.005	0.003
	Impala	Waterbuck	0.10	0.004	< 0.001
	Thomson’s gazelle	Grant’s gazelle	0.40	< 0.001	< 0.001
	*Impala*	*Lesser kudu*	*0.07*	*0.007*	*0.104*
Small NDVI	Zebra	Wildebeest	0.33	< 0.001	< 0.001
	Zebra	Eland	0.10	0.028	0.011
	Thomson’s gazelle	Grant’s gazelle	0.29	< 0.001	< 0.001
Medium NDVI	Zebra	Wildebeest	0.14	0.001	< 0.001
	Zebra	Waterbuck	0.05	0.015	0.003
	Thomson’s gazelle	Grant’s gazelle	0.65	< 0.001	< 0.001
Large NDVI	Zebra	Wildebeest	0.31	< 0.001	< 0.001
	Impala	Eland	0.11	0.009	0.019
	Thomson’s gazelle	Grant’s gazelle	0.32	< 0.001	0.003
	Thomson’s gazelle	Giraffe	0.21	0.009	0.045
	*Wildebeest*	*Thomson’s gazelle*	*0.20*	*0.006*	*0.079*
	*Wildebeest*	*Grant’s gazelle*	*0.18*	*0.024*	*0.385*
	*Wildebeest*	*Giraffe*	*0.19*	*0.013*	*0.074*

In some cases, the significance of the association strength can be explained by preferences for the same habitat types (lines highlighted in italics). For each scenario, all dyadic associations between the ten most frequently encountered species (giraffe, eland, zebra, wildebeest, waterbuck, lesser kudu, Grant’s gazelle, impala, Thomson’s gazelle, Kirk’s dik–dik) were tested. Only dyads with significant values are shown.

In sum, most dyadic associations were relatively constant across the observed environmental variability. However, inside the LHR, associations with giraffes were more pronounced (H6).

### Assortative mixing

To further untangle whether predation risk or food stress was the key driver for MSG formation, we tested if the composition of MSGs could be explained by similarities in susceptibility to lion predation or similarities in feeding habits. We found a significant relationship between the similarity in prey preferences by lions and the strength of associations in four of the five networks (inside the LHR: coefficient = 0.71, *p* = 0.048; outside the LHR: coefficient = 1.27, *p* = 0.022; small NDVI: coefficient = 1.24, *p* = 0.023; medium NDVI: coefficient = 1.41, *p* = 0.021), but not in the network under large NDVI conditions (coefficient = 0.50, *p* = 0.10). In contrast, we did not find a relationship between the similarity in feeding habits and the strength of associations in any of the networks (coefficients ranging from 0.10 to 0.70; *p*-values ranging from 0.33 to 0.46).

Assortative mixing provided evidence that predation risk but not necessarily food stress is a key driver for the composition of MSGs (H7).

## Discussion

Using field data collected along key environmental variables and employing generalized linear models and robust network analyses, we partitioned the relative effects of predation risk imposed by lions, primary productivity (approximated by using NDVI), and vegetation structure on the frequency and composition of MSGs in an East African herbivore community. In terms of predation risk, our results support the SGH both in quantitative and structural terms. MSGs occurred with a greater likelihood inside the LHR. Inside the LHR, herbivores were more likely to associate with heterospecifics that can dilute lion predation risk and with giraffe, a species likely to increase predator detection^33^. In terms of stress associated with primary productivity, we found mixed effects (in terms of strength and directionality) on species associations. More generally, our results indicate species-specific responses to different stressors, highlighting the importance of assessing the effects of environmental stressors on species interactions relative to a particular species.

While key results of our study, i.e. greater likelihood and structural changes of MSGs inside the LHR, are consistent with the SGH, we found variable effects of NDVI on the formation of MSGs. Before interpreting these results, a word of caution is in order about what we can infer from these observed effects. While the absence of potentially confounding predation pressure by other carnivore species^34^, a relatively stable composition of the herbivore community^35^, and our approach to control for habitat structure provided a suitable setting for this study, we cannot fully disentangle how the observed patterns are mediated by individual habitat choice patterns. Indeed, the effects of predation risk and NDVI on MSGs may partially mirror movement and habitat choice patterns of African savanna herbivores^23^. For example, movements of wildebeest in the Serengeti^36^, as well as wildebeest occurrence in MSGs in our study system (Fig. 2), were relatively unaffected by predation risk. In contrast, predation risk strongly mediated movement patterns^36^ and occurrence of zebra in MSGs (Fig. 2). Furthermore, while predation risk and primary productivity were not strongly collinear in our case (Table S1), dense or tall vegetation (which may coincide with large NDVI) may indeed be perceived as risky habitat. For example, Thomson’s gazelle, which typically prefer small NDVI conditions^23,37^ frequently associated with other species in areas of medium and large NDVI (Fig. 2), likely to reduce predation risk^38^.

Under the assumption that the differences in the strength and direction of NDVI on MSGs reflect species-specific habitat selection and perceptions and responses towards stressful conditions^32^, the U-shaped relationship between NDVI and species interactions at the community level may possibly align with predictions of the SGH. From the perspective of the herbivore community (and thus averaging across species), forage stress is likely minimized at medium NDVI levels because this is where energy intake is maximized for most species^25^ and where the sum of species-specific deviations from ideal forage conditions is minimized. While this provides a plausible explanation for the observed MSG-NDVI relationship at the community level, we acknowledge that teasing apart forage quality and quantity (which are often inversely related^4^) based on coarse, remotely sensed data, is ideally verified based on ground-truth data^36^.

Supporting predictions of the SGH, our results suggest that prey preferences by lions largely determine social affinities between species, thus providing evidence that species form MSGs to dilute predation risk. However, the insignificance of prey susceptibility under large NDVI conditions further corroborates the possibility that resource abundance mediates grouping patterns in herbivores, a result consistent with patch choice studies of herbivores suggesting that resource abundance can override predation risk^27,39,40^.

In line with this notion, dyadic affinities of most species did not differ between areas inside vs. outside the LHR (Table 3). Because failing to minimize predation risk (either by maximizing detection probability of predators or diluting mortality risk) has strong evolutionary ramifications^41,42^, social affinities are likely subject to strong selective pressure. This may explain why several dyadic affinities are sustained even in areas with low predation risk (Table 3).

The role of giraffes in social networks is an exception to this pattern. Though generally not showing high social affinities^6^, significant social affinities of giraffes with Thomson’s gazelles and eland (Table 3) and close ties with multiple other species (Fig. 3a) were restricted to areas inside the LHR (Table 3, Fig. 3b). Although it is difficult to infer a directional preference, we hypothesize that this pattern is driven by species seeking the proximity to giraffes. For example, behavioural studies suggest that zebras reduce individual vigilance in MSGs^43^, eavesdrop on cues produced by giraffes^33^, and thus benefit from proximity with giraffes beyond mere risk dilution^44^. Across an entire herbivore community, species with low vigilance rates were shown to be more likely to associate with species that elicit informative alarm calls^45^. Collectively, these studies suggest that the formation of MSGs is a key evolutionary response to increase predator detection.

While our results provide a coherent explanation that the observed patterns of animal networks align well with the SGH in terms of reducing stress associated with predation risk, animals in MSGs may compete for resources when associated with other species. While species have evolved multiple behavioural, morphological and physiological adaptations to coexist^24,46^ and theoretical models suggest that competition may not be a particular strong driver of interspecific associations^45^, we expect that the relative influence of facilitation versus interspecific competition may be context dependent. Especially at small NDVI, species in MSGs possibly compete for the same, restricted resources^47^. Testing the implied assumption that MSGs generally constitute positive interactions could be achieved with behavioural studies, measuring fitness relevant behaviour in mixed- and single-species groups along resource gradients^45^.

More generally, differences in species-specific grouping propensities (Fig. 2) indicate strong context dependence when assessing the impact of environmental variables on community structure and underscore the importance of choosing a species’ lens for disentangling relationships between grouping patterns of multiple organisms and environmental gradients^48,49^. Not accounting for species identity could bias relationships between grouping patterns and environmental variables (Fig. 2). Similarly, accounting for potentially confounding variables such as habitat structure (in this case: open habitats were positively associated with MSG formation) is crucial to estimate meaningful effect sizes of animal networks^50^.

While our framework allowed us to disentangle assortative processes underlying MSGs in a large mammal community along two crucial environmental variables, a caveat of our study is the effective sample size of one. We thus encourage studies to test the general validity of the outlined effects of multiple stressors on MSGs. Such analyses could also employ more nuanced proxies for human land use intensity^31^.

There is a growing body of evidence for shifts in interaction webs across ecosystems following declines of apex carnivores^51^. Our results and prior work on interspecific associations in African herbivores^4,5,7,9,45,52^, support the hypothesis that predation risk by African lions is a key selective force for the formation of MSGs. Although anti-predator adaptations in prey populations persist even when predators have been removed^53^, our analyses suggest nuanced impacts of lions on herbivore community structure. Therefore, we would expect shifts in herbivore community structure in lion-depleted environments^54,55^, such as fewer associations between giraffes and other species (Fig. 2, Table 3).

We also showed that animal networks are more complex at high and low NDVI. Given the close association between NDVI and precipitation^56^, this may be important for anticipated changes in climatic patterns across East Africa. As both rainfall amount and drought intensity are predicted to increase in East Africa^57^, herbivore communities are likely to be faced with increasingly stochastic environments with possible ramifications for the frequency and structure of MSGs.

The unifying pattern for predation risk and large NDVI values was that species co-occurrences were more evenly distributed as indicated by small values of the *Y*-measure inside the LHR and for large NDVI conditions (Fig. 4). This implies that the increase in the node strength was not caused by a few dominant associations, but by a generally greater propensity for MSGs inside the LHR and at large NDVI. Hence, the overall herbivore community seems to respond to variation in predation risk and primary productivity as a whole. Prima facie, these results seem to conflict with our results regarding significant dyadic associations. However, the significance of an association between a pair of species only means that the two species co-occurred more often than could be explained by random (or by preferences for habitat types) and not necessarily that the association strength of the two species was particularly large. While significant dyadic associations hint at certain behavioural patterns regarding a pair of species, our results concerning the network measures indicate differences in the general structure of MSGs.

Beyond dwindling lion populations and changes in rainfall patterns, elevated atmospheric CO_2_, livestock herding, and altered fire regimes are causing bush encroachment in East Africa^30,58^. Given the observed effects of habitat structure on dyadic associations, such vegetation shifts may not only alter relative abundances of browsing and grazing herbivores^59^, but may additionally affect herbivore community structure via habitat-dependent social affinities (Table 3).

Overall, our study provides support for the SGH and underscores the cascading and strong, yet indirect effect that apex predators exert on interactions among their prey. Mixed and non-linear relationships between NDVI and MSGs likely reflect the heterogeneity in stress perception and foraging strategies among species. Accounting for such species-specific effects of environmental stressors as well as considering that trade-offs between two different stressors may change with NDVI conditions is thus key for predicting species interactions in the Anthropocene.

## Methods

### Study area

We conducted this study in Manyara Ranch (MR), a 183 km^2^ non-fenced multiple use area located in the Tarangire ecosystem, northern Tanzania. The vegetation is dominated by *Acacia-Commiphora* savanna and the area receives an average of 651 mm of rainfall^60^. Ranch-owned livestock graze the area year-round. During the dry season, pastoralists from adjacent communities graze edge areas of MR. MR harbours a species-rich community of mammal species; most large mammal species occur at densities similar to neighbouring national parks^35^.

In MR, lions are the principal natural predators of herbivores during daytime. Based on a camera trap study^61^ conducted prior to this study (September–November 2014), spotted hyena (*Crocuta crocuta*) and striped hyena (*Hyeana hyeana*) occur on the ranch, but are almost exclusively active during night time. Leopards (*Panthera pardus*), cheetahs (*Acinonyx jubatus*) and African wild dogs (*Lycaon pictus*) were not detected during the camera trap survey.

### Data collection

This research was carried out with permission from relevant authorities (Tanzania Wildlife Research Institute; COSTECH permit #2014-324-ER-2013-191) and explicit consent from MR management. All methods were carried out in accordance with relevant guidelines and regulations and methods are reported in accordance with ARRIVE guidelines.

We conducted field work during the rainy season from February to April 2015. We deliberately chose this time of the year, because this is when MSGs are influenced by social affinities between species. During the dry season, the species-specific association patterns can largely be explained by the relative abundance of the species^6^. We collected mammal grouping data by driving along tracks of the ranch and recording any large (equal or greater than a dwarf mongoose *Helogale parvula*) mammal species within 200 m of the track, measured using a laser range finder (Table 1). We chose this threshold as this distance is similar to effective strip width estimates of distance sampling surveys (i.e. the area under the distance function from its left-truncation limit) for most species in this area^35^. To ensure that we detected all co-occurring species, we spent several minutes at each sighting and scanned the surroundings using 10 × 42 binoculars. Logistic regression analyses suggested that the likelihood of detecting species associations was not negatively affected by increasing distances. For each sighting, we recorded the GPS location, and counted the number of individuals. In line with previous research on MSGs^6^ and with the median threshold distance for defining animal groups^62^, we defined single species groups as individuals within 50 m of conspecifics and MSGs as groups of two or more heterospecifics within 50 m of one another. We alternated daily routes to avoid pseudo replications; observations from the same location are separated by at least 24 h. We assessed explanatory variables (lion home range, NDVI, and a proxy for human activity) after the completion of fieldwork, and thus included a blinding element during fieldwork.

### Explanatory variables

To assess the spatial dimension of lion predation risk, we estimated the home range of lions (Fig. 1). We utilized lion occurrence data (n = 23 observations) that were collected from 2010 to 2015. Considering the limited sample size, we modelled the extent of the lion home range (hereafter LHR) using the minimum convex polygon method with 85% isopleths. From 2015 to 2019, subsequent opportunistic lion observations (n = 3) occurred within the delineated LHR; nevertheless we cannot exclude the possibility that the resident lions used areas outside the estimated home range or that nomadic lions traversed the area. We plotted mammal sightings and LHR in GIS software (ArcGIS) and extracted corresponding spatial data for each animal group.

As a proxy for habitat productivity, we extracted time matched values of the Normalized Difference Vegetation Index (NDVI). We used the NDVI layer from the MODIS Terra vegetation index product (MODI3Q1 V6). This layer “is computed from atmospherically corrected bi-directional surface reflectances that have been masked for water, clouds, heavy aerosols, and cloud shadows”^63^. For each field observation, we constructed a 200 m buffer around the observation location (because sightings were up to a maximum of 200 m distant from the recorded GPS locations), and calculated the average NDVI of intersecting cells, weighted by area. We used NDVI values from the date closest to each field observation; differences between observations and imagery ranged from 0 to 8 days. We binned these NDVI scores in three equal sized classes (small, medium, and large using 0.33 and 0.67 percentiles; Fig. S1a). Categorization of this variable was necessary to construct a separate animal network for each scenario. Also, the distribution of the MSGs relative to the NDVI scores was difficult to describe by a continuous function (Fig. S1b).

To control for structural differences in habitat, we assigned one of four habitat types based on vegetation physiognomy (grassland, open bushland, bushland, riverine) to each mammal sighting during field work.

To test if human activity mediated MSGs, we categorized locations into areas with low and high human activity. Observations in the core of the ranch (> 2 km from the outer ranch boundary) were assumed to have low human activity. Observations located in the edge area of the ranch (≤ 2 km from the ranch boundary) were assumed to experience greater human activity (Fig. 1). We chose this 2 km threshold because grazing by community cattle is restricted to this 2 km zone.

To assess collinearity across explanatory variables we calculated Cramér’s V scores; association strengths across pairs of hypothesized variables were moderate (V: 0.13 to 0.34; Table S1).

### Analytical framework

We employed a hierarchical procedure and initially tested the occurrence of mixed—(coded as 1) versus single-species groups (0) in a logistic regression model for the animal community and separately for the six most frequently encountered species: giraffe, zebra, wildebeest, Grant’s gazelle, impala, and Thomson’s gazelle. We estimated effect sizes by estimating the odds ratios (Fig. 2). Based on the strength of relationships (threshold p = 0.05) with the hypothesized explanatory variables (Table S2), we investigated the networks across the observed variation in NDVI and LHR conditions (the human activity variable did not produce a significant signal; the habitat structure variable was kept as control variable). We performed logistic regression analyses and visualization of most results using R 3.60^64^.

### Animal networks

To assess structural properties of the MSGs and to provide evidence for the assumption that MSGs represent positive interactions, we conducted network analyses. First, we constructed weighted networks with the species as nodes and edges between the nodes *i* and *j*, if species *i* and *j* co-occurred in at least one group. For our network analyses we require edge weights that represent association strengths. Also, it is important that this relation is symmetric, i.e. the association strength between species *i* and *j* must be the same as that between *j* and *i*. As a suitable and simple solution we computed the edge weights *w*_*i,j*_ using Dice’s index^65^:

$$w_{i,j} = { 2}N_{i,j} /\left( {N_{i} + N_{j} } \right),$$

where *N*_*i*_, *N*_*j*_, and *N*_*i,j*_ are the numbers of groups with species *i*, species *j*, and both *i* and *j*, respectively. Dice’s index measures the strength of association between two species *i* and *j* by computing the ratio of the number of their observed co-occurrences and the number of their potential co-occurrences, given the number of times each of the two species was observed. Its values range from 0 (species *i* and *j* were never observed together in the same group) to 1 (species *i* and *j* were observed together in all groups, in which one of the two species occurred).

According to the results of the logistic regressions (significant effects of LHR and NDVI on the likelihood of MSGs), we created five networks, two from groups inside and outside LHR, respectively, and three from groups in areas with small, medium and large NDVI values (thresholds: 0.33 and 0.67 percentiles of realized values), respectively. Although habitat type was also significantly associated with mixed species groups, we did not construct separate networks due to a lack of specific hypotheses. However, we took potential preferences for habitat types into account in our randomisation tests (see below). We included only those 15 species that occurred in all five networks (species listed in Table 1, excluding gerenuk, baboon, and buffalo), so that the analysed networks had the same numbers of nodes.

To visualise the network structures we constructed condensed network figures and highlighted focal species for which the explanatory variables yielded a significant signal.

We compared the networks inside and outside the LHR, and the networks for the three NDVI conditions. The observations outside the LHR were subsampled to match the number of observations inside the LHR. We chose 3 common, well-established node-based network measures widely used in animal social network analysis^66^ that have a biologically intuitive meaning regarding social relationships: the node strength, the *Y*-measure, and the weighted clustering coefficient. These measures were chosen to cover different aspects of networks but they partly also complement each other (in particular the node strength and the *Y*-measure). The node strength of node *i* measures the total weight of all edges connected to *i*. In our networks, high node strengths can either signify a large number of neighbouring nodes or strong connections to neighbouring nodes. The *Y*-measure of node *i* is the sum of squares of the normalized edge weights of *i* (edge weights divided by the node strength of *i*) and quantifies the disparity in the association strengths of a node. A small value means that a species is connected to other species with similar association strengths, while a large value means that some associations dominate the node strength. The weighted clustering coefficient of node *i* quantifies the extent to which neighbours of *i* are themselves neighbours and thus characterizes subsets of highly connected species in a network.

To perform the network comparisons, we subsampled our data to create 3 independent replicates for each of the 5 networks (Table S3), computed the values of the network measures and aggregated these values for each network by computing the mean of the values of our six focal species. Thus, for each of our 5 scenarios (inside and outside the LHR, and small, medium, and large NDVI) we obtained 3 aggregated values for each of the 3 network measures. A simulation study based on random networks with the same characteristics as our observed networks showed that the distributions of these aggregated values did not substantially deviate from a log-normal distribution (Fig. S2). Therefore, we used a two-tailed Welch two sample t-test on the logarithms of the aggregated network measures for our comparisons.

### Randomisation tests

Because of the dependent nature of association strengths in a network, we performed randomisation tests for analysing species-specific association patterns and assortative mixing. To analyse species-specific association patterns, we placed all sightings of single- and mixed-species groups in a species occurrence matrix, where a “1” in row *i* of column *j* denotes that species *j* was present in group *i*, while a “0” marks its absence. We adopted as a null model all species occurrence matrices with the same row and column sums as the observed one to be equally likely and used the method of Besag and Clifford^67^ to randomise the observed group compositions. To assess if results could be explained by utilisation of the same habitats, we also kept constant the numbers of occurrences of each species per habitat type. We did this because habitat type significantly affected the formation of MSGs. We used as a test statistic the number of co-occurrences of species, which is equivalent to using Dice’s index, because the randomisation keeps constant the number of occurrences of each species. We only tested those pairs of species that co-occurred more than two times.

To test whether similarity in susceptibility to lion predation or information about food availability were the key driver for MSG composition, we used a logistic regression model. As the response variable, we used the association strength (Dice’s index) between pairs of species and included the two explanatory variables: similarity in prey preferences by lions and similarity in food preferences. To express the similarity in prey preference by lions, we computed the absolute value of the difference of the values of Jacobs’ index^68^. This index quantifies the prey preference (positive values) or avoidance (negative values) by lions (Table 1). To express the similarity in feeding habits we computed the absolute value of the difference of the proportion of monocot consumption (Table 1). Both measures of similarity were multiplied by − 1 so that higher values indicated stronger similarity. We tested the significance of coefficient values by performing a node label permutation with 10^4^ steps. In each step we shuffled the species’ attributes (Jacobs’ index and monocot consumption), computed the explanatory variables, and fitted the model. We hypothesised that the association strength increased with the similarity indices and regarded an observed coefficient value as significant, if it was among the 5% largest values.

## Supplementary Information

Supplementary Information.

## Data Availability

Data are available from the Göttingen Research Online repository: 10.25625/KRTSLI.
