# Supplementary material for: Refining the stress gradient hypothesis for mixed species groups of African mammals
Source: Sci Rep. 2022 Oct 21;12:17715. doi: 10.1038/s41598-022-22593-3 (PMC9587046; doi:10.1038/s41598-022-22593-3)

**Supplementary material for:**

**Refining the stress gradient hypothesis for mixed species groups of African mammals**

Christian Kiffner^1,2*^, Diana Boyle^3^, Kristen Denninger-Snyder^4,5^, Bernard M. Kissui^2^, Matthias Waltert^3^ & Stefan Krause^6^

**Table S1.** Cramér’s V scores of associations between hypothesized variables (Habitat: habitat categories with 4 levels; NDVI: Normalized Difference Vegetation Index values binned in three categories; LHR: location inside/outside of lion home range; human activity: indicated by location in the “core” [≥ 2 km from boundary] or “edge” [< 2 km from boundary] of the ranch) related to mixed species groups in Manyara Ranch, northern Tanzania.

|  | LHR | NDVI | Habitat |
| --- | --- | --- | --- |
| NDVI | 0.34 |  |  |
| Habitat | 0.23 | 0.21 |  |
| Human activity | 0.29 | 0.13 | 0.22 |

**Table S2.** Regression coefficients, associated 95% confidence intervals and test-statistics of logistic regression models testing the likelihood of mixed species group (MSG) occurrence. We ran models for the entire wildlife species assemblage and separate models for giraffe, zebra, wildebeest, Grant’s gazelle, impala, and Thomson’s gazelle. As explanatory variables, we considered habitat type (three- or four-level categorical variable), vegetation productivity (NDVI; three-level categorical variable), and location of the group in relation to the lion home range (two-level categorical variable). For the entire species assemblage, we also included the location of the group within the ranch (core vs. edge). Because this variable did not produce a significant signal, we did not include it in the species-specific models. Significant associations (p≤0.05) are highlighted in bold.

|  |  | **Estimate** | **Std. Error** | **z-value** | **p-value** |
| --- | --- | --- | --- | --- | --- |
| **All species** | **Intercept** | **-1.893 (-2.351; -1.459)** | **0.227** | **-8.329** | **<0.001** |
| **(n = 951)** | Grassland (vs. bushland) | 0.201 (-0.258; 0.654) | 0.232 | 0.865 | 0.387 |
|  | **Open bushland (vs. bushland)** | **0.402 (0.036; 0.773)** | **0.188** | **2.141** | **0.032** |
|  | Riverine (vs. bushland) | -0.617 (-1.414; 0.095) | 0.381 | -1.617 | 0.106 |
|  | **NDVI small vs. medium)** | **0.600 (0.202; 1.006)** | **0.205** | **-2.931** | **0.003** |
|  | **NDVI (large vs. medium)** | **0.493 (0.083; 0.910)** | **0.211** | **2.342** | **0.019** |
|  | **Lion home range (inside vs. outside)** | **0.526 (0.163; 0.890)** | **0.185** | **2.842** | **0.004** |
|  | Ranch (core vs. edge) | -0.012 (-0.387; 0.369) | 0.193 | -0.063 | 0.949 |
|  |  |  |  |  |  |
| **Giraffe** | **Intercept** | **-1.108 (-1.920; -0.354)** | **0.397** | **-2.791** | **0.005** |
| **(n = 128)** | Grassland (vs. bushland) | 0.919 (-0.345; 2.264) | 0.655 | 1.404 | 0.160 |
|  | Open bushland (vs. bushland) | 0.458 (-0.364; 1.295) | 0.421 | 1.087 | 0.277 |
|  | Riverine (vs. bushland) | 16.558 (-279.861; NA) | 1455.398 | 0.011 | 0.991 |
|  | NDVI (small vs. medium) | -0.056 (-1.064; 0.942) | 0.509 | -0.109 | 0.913 |
|  | NDVI (large vs. medium) | 0.116 (-0.806; 1.028) | 0.465 | 0.249 | 0.803 |
|  | **Lion home range (inside vs. outside)** | **1.166 (0.376; 1.983)** | **0.408** | **2.859** | **0.004** |
|  |  |  |  |  |  |
| **Zebra** | **Intercept** | **-1.374 (-1.832; -0.941)** | **0.227** | **-6.061** | **<0.001** |
| **(n = 498)** | Grassland (vs. bushland) | -0.360 (-0.908; 0.181) | 0.277 | -1.298 | 0.194 |
|  | Open bushland (vs. bushland) | 0.008 (-0.467; 0.484) | 0.242 | 0.032 | 0.974 |
|  | Riverine (vs. bushland) | 0.584 (-0.483; 1.637) | 0.534 | 1.095 | 0.274 |
|  | **NDVI (small vs. medium)** | **0.814 (0.335; 1.306 )** | **0.247** | **3.294** | **0.001** |
|  | **NDVI (large vs. medium)** | **0.811 (0.283; 1.346)** | **0.271** | **2.998** | **0.003** |
|  | **Lion home range (inside vs. outside)** | **0.527 (0.091; 0.961)** | **0.222** | **2.376** | **0.018** |
|  |  |  |  |  |  |
| **Wildebeest** | Intercept | 0.875 (-0.254; 2.142) | 0.601 | 1.457 | 0.145 |
| **(n = 118)** | Grassland (vs. bushland) | 0.467 (-0.947; 1.910) | 0.716 | 0.652 | 0.515 |
|  | Open bushland (vs. bushland) | -0.140 (-1.490; 1.140) | 0.663 | -0.212 | 0.832 |
|  | Riverine (vs. bushland) | -1.077 (-2.883; 0.698) | 0.896 | -1.203 | 0.229 |
|  | NDVI (small vs. medium) | 0.711 (-0.575; -1.982) | 0.644 | -1.104 | 0.270 |
|  | NDVI (large vs. medium) | 0.414 (-0.829; 1.625) | 0.618 | 0.670 | 0.503 |
|  | Lion home range (inside vs. outside) | -0.051 (-1.068; 0.985) | 0.519 | -0.098 | 0.922 |
|  |  |  |  |  |  |
| **Grant's gazelle** | Intercept | -0.441 (-1.577; 0.654) | 0.560 | -0.787 | 0.431 |
| **(n = 96)** | Grassland (vs. bushland) | 0.715 (-0.583; 2.044) | 0.664 | 1.077 | 0.282 |
|  | **Open bushland (vs. bushland)** | **1.240 (0.162; 2.385)** | **0.562** | **2.206** | **0.027** |
|  | NDVI (small vs. medium) | -0.453 (-1.641; 0.687) | 0.589 | 0.769 | 0.442 |
|  | NDVI (large vs. medium) | -0.670 (-1.952; 0.529) | 0.627 | -1.068 | 0.285 |
|  | **Lion home range (inside vs. outside)** | **1.176 (0.095; 2.353)** | **0.570** | **2.064** | **0.039** |
|  |  |  |  |  |  |
| **Impala** | **Intercept** | **-1.807 (-2.652; -1.055)** | **0.405** | **-4.461** | **<0.001** |
| **(n = 137)** | Grassland (vs. bushland) | 0.518 (-2.793; 3.834) | 1.477 | 0.351 | 0.726 |
|  | **Open bushland (vs. bushland)** | **0.820 (-0.002; 1.650)** | **0.419** | **1.957** | **0.050** |
|  | Riverine (vs. bushland) | -0.019 (-1.773; 1.533) | 0.823 | -0.023 | 0.981 |
|  | NDVI (small vs. medium) | 0.575 (-0.583; 1.729) | 0.584 | 0.983 | 0.326 |
|  | NDVI (large vs. medium) | 0.577 (-0.276; 1.453) | 0.438 | 1.316 | 0.188 |
|  | **Lion home range (inside vs. outside)** | **1.001 (0.211; 1.807)** | **0.405** | **2.470** | **0.014** |
|  |  |  |  |  |  |
| **Thomson‘s gazelle** | Intercept | 2.420 (0.326; 5.722) | 1. 293 | 1.871 | 0.061 |
| **(n = 85)** | Grassland (vs. bushland) | -0.148 (-2.411; 1.872) | 1.054 | -0.141 | 0.888 |
|  | Open bushland (vs. bushland) | 0.953 (-1.233; 2.924) | 1.017 | 0.937 | 0.349 |
|  | **NDVI (small vs. medium)** | **-3.144 (-6.278; -1.106;)** | **1.229** | **-2.558** | **0.011** |
|  | NDVI (large vs. medium) | -0.858 (-4.013; 1.347) | 1.261 | -0.680 | 0.497 |
|  | Lion home range (inside vs. outside) | -0.464 (-2.623; 1.451) | 0.994 | -0.466 | 0.641 |

**Table S3.** Creation of independent network samples. For each of the 5 networks (inside and outside the LHR; low, medium, and high NDVI conditions) we created 3 independent replicates. To do this, we randomly split the underlying set of observations of each network into 3 independent subsets of equal size. Using an optimisation procedure, we made sure that all observations of the same day were put into the same subset (thus avoiding potential dependencies between the subsets) and that all 3 subsets were based on the same number of days and contained equal numbers of observations, differing at most by 1. Finally, we constructed networks from each subset. The table below summarizes the subnetworks and associated measures used for the network comparisons.

| Network | Subnetwork | Number of observation days | Number of groups | Node strength | Y measure | Clustering coefficient |
| --- | --- | --- | --- | --- | --- | --- |
|  |  |  |  |  |  |  |
| Inside lion home range | 1 | 8 | 111 | 1.153 | 0.201 | 0.145 |
|  | 2 | 7 | 111 | 0.901 | 0.175 | 0.101 |
|  | 3 | 7 | 111 | 1.037 | 0.208 | 0.117 |
| Outside lion home range | 1 | 12 | 111 | 0.382 | 0.542 | 0.051 |
|  | 2 | 11 | 111 | 0.508 | 0.356 | 0.062 |
|  | 3 | 11 | 111 | 0.458 | 0.470 | 0.053 |
| Low NDVI | 1 | 9 | 106 | 0.391 | 0.522 | 0.060 |
|  | 2 | 9 | 105 | 0.505 | 0.379 | 0.055 |
|  | 3 | 9 | 105 | 0.677 | 0.312 | 0.090 |
| Medium NDVI | 1 | 11 | 105 | 0.743 | 0.307 | 0.114 |
|  | 2 | 11 | 105 | 0.506 | 0.523 | 0.075 |
|  | 3 | 10 | 104 | 0.508 | 0.598 | 0.036 |
| High NDVI | 1 | 7 | 105 | 1.074 | 0.169 | 0.106 |
|  | 2 | 7 | 105 | 0.765 | 0.194 | 0.073 |
|  | 3 | 6 | 106 | 0.968 | 0.252 | 0.143 |

**Figure S1.** Frequency of mixed species groups (MSGs) according to a) three and b) five equal-sized classes of the NDVI values. The bins were based on 0.33 and 0.67 percentiles for a) and 0.2, 0.4, 0.6, and 0.8 percentiles for b).

**
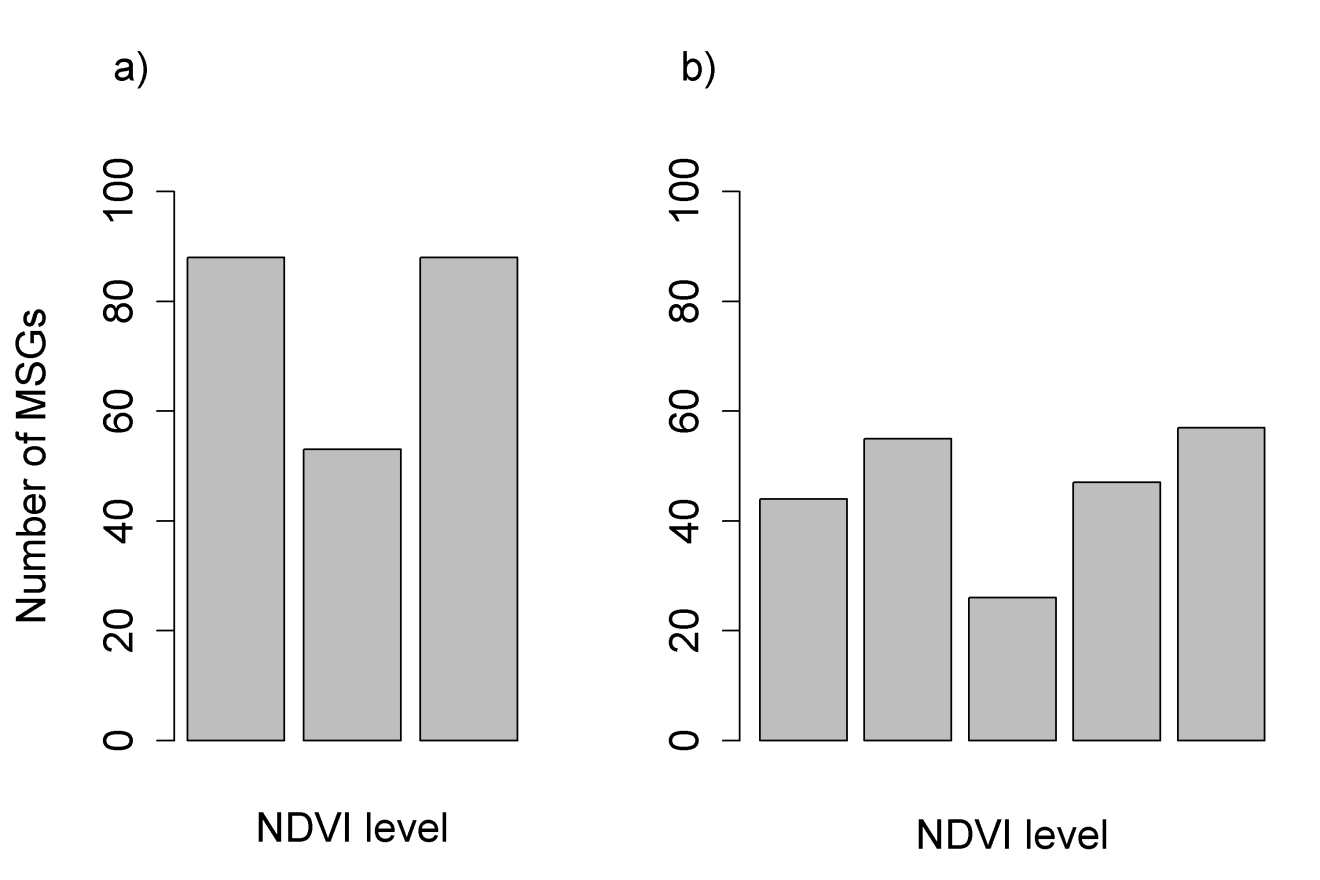
**

**Figure S2.** Normal quantile-quantile plots of the logarithms of aggregated values of a) node strength, b) Y-measure, and c) weighted clustering coefficient of 1000 random networks with similar characteristics as the observed ones. The networks were constructed from simulated “observations” of mixed species groups, where the number of groups, the number of species, the frequencies of the species, and the mean number of species per group were similar to the actual observations.


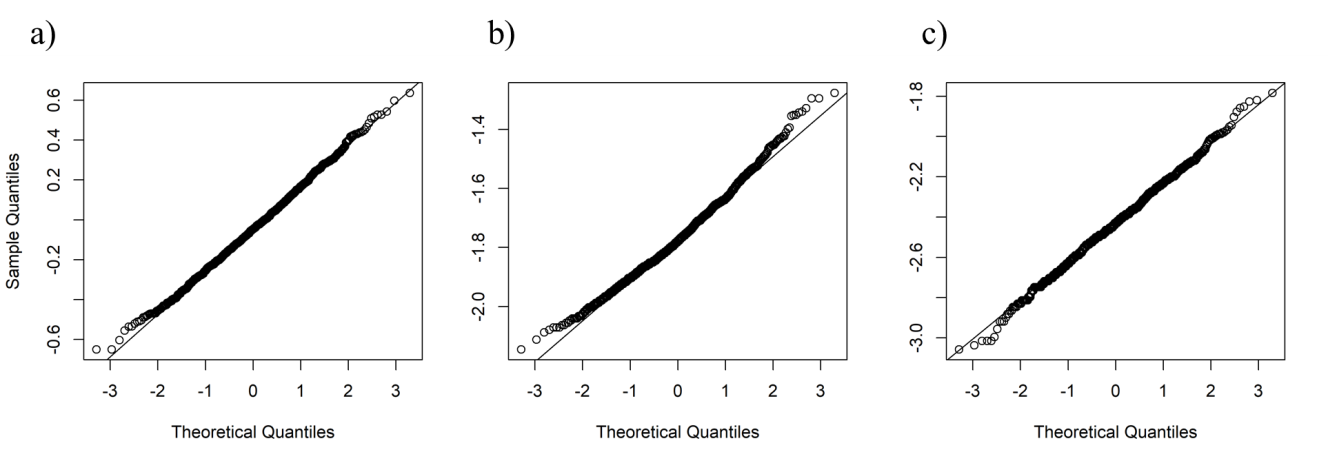

Supplement: Supplementary file 1 — Supplementary Information. [file 41598_2022_22593_MOESM1_ESM.docx]
